# The Oxford Companion to Medicine

**Published:** 1987-11

**Authors:** J. D. Davies

**Affiliations:** University of Bristol


### Book Review

**THE OXFORD COMPANION TO MEDICINE**

**Eds. J. Walton, P. B. Beeson and the late R. B. Scott**

**Oxford University Press, Oxford, 1986**

**pp. 1524+xxii, hardback.**

**2 volumes**

**ISBN 0 19 261191 7 (set).**

**Price £65.00**

The title of this book had put me off until your editor said that he thought it was worth reading. Michael Wilson was quite right. This is no potted synopsis of Music, Theatre or anything else for the uninitiated. It is a solid 1500 pages of worthwhile information. The original intended readership was 'practitioners of medicine whatever their speciality'. So it clearly is. It is less idiosyncratic than others in the area. However, where else are you likely to find a working definition of *Vibromassage* amongst the more serious entries of a discursive encyclopaedia of medicine in general?

We all lose touch in other fields, without knowing it. Personally I regret the local dissolution of the combined Bristol Final MB vivas involving pathologists, ophthalmologists, ENT surgeons and Community Physicians, all on the same table for a day. It mattered little if some of the more central Bristol centres couldn't even provide sandwiches for lunch! We all learnt a lot from each other, and not least from the candidates. Now, we will have to make do with books like this one.

Petty parochial issues aside, how does this particular Oxford Companion match up to its predecessors? First, let me say that it is a serious and comprehensive book. There are numerous helpful essays on the current state of many different topics. Recognised clinical specialities, the General Medical Council, Law and Medicine in the UK and USA, art and medicine, Notable Patients (no, not the particularly trying ones you see every month in the follow-up clinic), the English Coroners' System, and Women in Medicine are the subject of some of these excellent extended reviews.

Brief biographical notes, though only of the dead, are generally worthwhile if only because they are not readily at hand elsewhere. These tend to be 'strictly' factual, and often discretely bypass the more interesting eccentricities of their subjects. They certainly lack the fun of Bailey and Bishop's *Notable Names.* None the less, some of them (e.g. that of Johannes Müller 1801–1858) are not just repetitions of the usual views.

So far so good. What else is there behind the respectable façade of the Oxford University Press? Are there any Gallicisms to make purchase really worthwhile? Oh yes, that I promise. For instance, page 10 fell open with a table headed 'A Moral and Physical Thermometer'. Your reviewer was horrified to see what might have lain in store for him, had he not been a bit stronger minded, in this section on Addiction. Good heavens, instead of *Health and Wealth* there lay just round the bend *Horse Racing, Sore and Swelled Legs,* and worse still *The Hospital.* Who knows what your own personal vices are? Maybe it's better not to consult this learned tome. Just for the record, Hogarth's *Rake's Progress* is included as well.

What else? First, good marks to the Companion for a modern definition of *'biopsy'.* Very bad marks for the 2nd MB fail-level definition of a granuloma (p. 502), and also for being bullied by Bernard Lennox on p. 389.

The Bristol Royal Infirmary? Yes, it's there. Sadly, history has a habit of repeating itself. The Oxford Companion, with independent historical perspective, feels able to say about the BRI: 'Like similar institutions it soon became inadequate for its purposes, so a new building was raised in 1784, completed in 1814, and still remains in use'. I am sure that it is not only your reviewer who bites his lip hard to say no more.

This book, though, is well worth buying. There is much to learn from it.

J. D. Davies

University of Bristol

